# Beyond the Label: Psychometric Validation of the Persian Stigma and Self‐Stigma Scales: A Methodological Study

**DOI:** 10.1002/hsr2.72375

**Published:** 2026-05-05

**Authors:** Hadi Tehrani, Alireza Jafari, Mahbobeh Nejatian, Maliheh Gholamzadeh, Fatemehzahra Naddafi

**Affiliations:** ^1^ Social Determinants of Health Research Center Mashhad University of Medical Sciences Mashhad Iran; ^2^ Department of Health Education and Health Promotion, School of Health Mashhad University of Medical Sciences Mashhad Iran; ^3^ Department of Health Education and Health Promotion, School of Health, Nursing Research Center Gonabad University of Medical Sciences Gonabad Iran; ^4^ Social Development and Health Promotion Research Center Gonabad University of Medical Sciences Gonabad Iran; ^5^ Department of Medical Surgical Nursing, Faculty of Nursing Gonabad University of Medical Sciences Gonabad Iran; ^6^ Department of Geriatric Health, Faculty of Health Tabriz University of Medical Sciences Tabriz Iran; ^7^ Student Research Committee Tabriz University of Medical Sciences Tabriz Iran

**Keywords:** mental health, mental illness, psychometric, self‐stigma, stigma, validity

## Abstract

**Background and Aims:**

Mental health disorders are becoming increasingly prevalent, making early detection and intervention crucial. However, stigma remains a significant barrier to accessing mental healthcare services. This study aimed to translate and culturally adapt the Persian version of the Stigma and Self‐Stigma Scales (SASS) to assess public attitudes toward mental health issues.

**Methods:**

This methodological study was conducted from August to December 2023 with a sample of 962 Iranian participants. The research evaluated the psychometric properties of the SASS. The assessment included content validity, face validity, and construct validity (confirmatory factor analysis (CFA)) and reliability indices.

**Results:**

The Scale‐Content Validity Index (S‐CVI/Ave) for all SASS items was excellent at 0.963. Following psychometric evaluation, seven items were eliminated. Confirmatory factor analysis demonstrated adequate model fit, with key indices including: χ²/df = 3.325, RMSEA = 0.049, CFI = 0.912, GFI = 0.920, IFI = 0.912, PNFI = 0.777, PGFI = 0.759, TLI = 0.901, and AGFI = 0.903. The final validated scale comprised 29 items organized into six distinct factors: Stigma toward others (4 items), Social distance (6 items), Anticipated stigma (6 items), Self‐stigma (6 items), Avoidant coping (3 items), Help‐seeking behaviors (4 items). The Persian SASS demonstrated overall acceptable reliability across its subscales (Cronbach's α = 0.911, McDonald's ω = 0.925) and acceptable test‐retest reliability (ICC = 0.677) except one of the subscales (help‐seeking behaviours), which had inadequate reliability and is recommended not to be used.

**Conclusion:**

The Persian adaptation of the SASS was successfully validated as a 29‐item instrument comprising six clinically relevant dimensions. This culturally adapted tool demonstrates acceptable psychometric properties for comprehensive stigma assessment in Persian‐speaking populations. Its multidimensional structure makes it particularly valuable for: Baseline evaluation prior to anti‐stigma interventions, Measuring specific stigma components in clinical and research settings and Tracking changes in public attitudes toward mental health.

**Abbreviations**
AGFI: adjusted goodness of fit Index
CFA: confirmatory factor analysis
CFI: comparative fit index
df: Degree of freedom
GFI: goodness of fit index
IFI: incremental fit index
PGFI: Parsimony goodness‐of‐fit index
PNFI: parsimonious normed fit index
RMSEA: root mean square error of approximation
SASS: The Stigma and Self‐Stigma scales
TLI: Tucker Lewis Index, Parsimony comparative fit index
x2: Chi‐square ratio to

## 1 Introduction

Mental illnesses represent a significant global public health challenge [1] and a common concern for both current and future generations [2]. Globally, approximately one billion people are affected by mental disorders [3]. Furthermore, annual global prevalence estimates indicate that approximately 17.6% of adults experience mental disorders, including depression, anxiety, and post‐traumatic stress disorder [4, 5]. In recent years, Iran has faced an increase in the prevalence of mental illnesses. A systematic review study conducted in 2020 showed that 31.03% of Iranian people, suffer from mental illnesses [6]. Mental illnesses are associated with significant impairments in professional and social functioning, as well as an increased economic burden on society. Moreover, they contribute to elevated rates of suicide, morbidity, and mortality [7, 8, 9, 10].

Early intervention by seeking help from mental health specialists is crucial. It plays a pivotal role in improving quality of life and social functioning, reducing the overall burden of mental illnesses, preventing relapse, and mitigating associated social and personal costs [9]. However, a significant treatment gap exists, as many individuals with mental disorders do not seek professional help. Consequently, less than 30% of those affected receive the necessary treatment [11].

Stigma surrounding mental illness constitutes a major obstacle to seeking help [12]. The stigma and labeling associated with mental illness often deter individuals from accessing mental health services, despite their availability [13]. As a result, the stigma of mental illness is an important barrier against seeking help and, as a potential cause, causes people's reluctance to seek help [12, 14]. Stigma literally means “a sign of shame, infamy and scandal” [15, 16]. Corrigan conceptualizes stigma as marks that denote a ‘spoiled identity,’ noting that these marks can be either overt—such as physical characteristics that lead to racism or sexism ‐ or concealed in nature [17]. The stigma of mental illness is specifically defined as scandal, social discredit, or social disapproval of people with mental health problems [18].

Stigma related to mental illness is widely recognized as a multidimensional phenomenon, comprising critical facets like public stigma, self‐stigma, and anticipated stigma [19, 20, 21]. Public stigma is the negative attitude, prejudice, and discrimination of society members toward people with mental illnesses [20]. Self‐stigma, or internalized stigma, occurs when individuals with mental illness endorse negative public stereotypes and turn them against themselves [18]. Anticipated stigma also denotes to the expectations that a person has about experiencing stigma in the future [22].

The stigma of mental illness unfortunately occurs in all ethnicities, religions, ages, and social and economic conditions. Statistics showed that in different societies, 22.5% to 97.4% of people with mental illnesses suffer from self‐stigma [23, 24, 25, 26]. In addition, a study in Tehran showed that approximately 40% of people with mental disorders experience degrees of severe to moderate stigma [27]. Consequently, stigma represents a pervasive global challenge that adversely affects quality of life, complicates the treatment process, exacerbates the course of illness, and undermines recovery outcomes [18, 23]. Therefore, implementing effective interventions to reduce mental health stigma is a public health priority [28].

Numerous instruments have been developed to assess stigma intensity and to evaluate and monitor the effectiveness of anti‐stigma interventions [29]. Corrigan et al., developed and psychometrically evaluated the Self‐Stigma of Mental Illness Scale (SSMIS). This scale with 40 items was used to examine self‐stigma in a population of patients with mental disorders [30, 31]. Day et al., developed the stigma of mental illness scale. This scale with 28 items, designed to assess individuals' attitudes toward people with mental illness [32]. Barney et al., developed and psychometrically evaluated the Self‐Stigma of Depression Scale (SSDS). This scale had 16 items and has been used both in the general population and in the patient population [33]. Ranjbar Karmani et al., investigated the psychometric properties of the social distance scale among Iranian people and this questionnaire included 7 items and was used in the population of mental health professionals [34]. Hedayati et al., examined the psychometric properties of the Self‐stigma of Seeking Help (SSOSH) Scale in Iran. However, this instrument only considers the help‐seeking dimension. Also, among the different forms of stigma, it only measures self‐stigma [35]. Gohari et al., evaluated the characteristics of the Stig‐9 questionnaire in Iran. However, this questionnaire was also designed only for perceived stigma of mentally ill patients [36].

Another available tool is the Stigma and Self‐Stigma Scales (SASS). This scale was presented in English by Docksey et al., in 2022 [21]. This scale includes 36 items and 6 factors of Self‐stigma, Stigma to others, Avoidant coping, Anticipated stigma, social distance and Help‐seeking behaviors. This tool has several advantages over other presented tools: First, unlike most stigma tools that only examine a specific aspect of stigma, it is comprehensive and evaluates different types of stigma. Second, some tools are designed for specific mental disorders (e.g. depression), while examining the stigma variables of mental illness in general brings a better understanding. In sum, Unlike existing instruments such as the 40‐item Self‐Stigma of Mental Illness Scale (SSMIS), which measures only self‐stigma, the SASS simultaneously assesses six distinct dimensions of both public stigma and self‐stigma with a notably lower number of items. This multidimensional nature, combined with its brevity, makes it a uniquely efficient and comprehensive tool. Furthermore, its validation in a community cohort confirms its suitability for diverse populations, from the general public to clinical settings, allowing for a more holistic investigation of the stigma continuum [21]. As a result, SASS is a more appropriate tool for measuring the stigma of mental illnesses than other tools. According to the studies, this tool has not been translated and psychometrically tested in Iranian society. Therefore, given the rising prevalence of mental illnesses and the limited availability of validated Persian stigma scales suitable for both general and clinical populations [37], this study was conducted with the aim of translating and psychometrically analyzing this scale in a Community Cohort of Iranian people.

## 2 Methods

### 2.1 Study Design and Sampling Method

The present study is a methodological study that was conducted from August to December 2023. to assess the validity and reliability of SASS among the general population of Gonabad, Iran (Gonabad is a relatively small and traditional city in terms of culture and ethnicity, and psychological stigma may be higher in this city, which is why it was chosen as the sampling location, although it is recommended that other researchers in other parts of Iran evaluate this questionnaire). The sample consisted of people from the general population of Gonabad who were selected using the proportional stratified random method. First, each health center in Gonabad was considered as a stratum, and then sampling was conducted according to the population covered by each center. In this study, both online and face‐to‐face methods were used to collect data. After selecting the samples, the purpose of the study was explained to the participants and informed consent was obtained. Then, the questionnaires were delivered directly to the individual at the health center or at home and completed as a self‐report. If it was not possible to visit, the questionnaire was sent to the individual online. The inclusion criteria included age over 15 years, informed consent, living in Gonabad city for at least 1 year, and the absence of cognitive problems (Using the individual's health record at health centers or reports from family and relatives).

### 2.2 Sample Size

Given that there is no specific sample size formula in psychometric studies, sample size of 500 and more is good for testing the CFA [38, 39]. In this study, 962 participants were considered.

#### 2.2.1 Instruments

##### 2.2.1.1 Demographic Section

In this section, information including marital status, education, sex, occupation, age, and receipt of mental health information (by self‐reported assessment) was assessed.

##### 2.2.1.2 The Stigma and Self‐Stigma Scales (SASS)

This instrument was designed by Docksey et al., in 2022, and its psychometric properties were investigated [21]. The original version of this questionnaire is in English and contains 36 items that are placed in 6 factors of Self‐stigma, Stigma to others, Avoidant coping, social distance, Help‐seeking behaviors and Anticipated stigma. Each factor has 6 items. In addition, this questionnaire includes 6 items under the title of social desirability, which are designed to measure social desirability bias. Answers to these questions are measured using a 5‐option Likert scale (strongly disagree = 0 to strongly agree = 4). Finally, after deleted of 7 items (Due to low factor loadings less than 0.3) in this study, the total score of SASS is 0 to 116 and the higher score show more stigma.

##### 2.2.1.3 Translation Process

Written permission was obtained after correspondence with the author of the instrument. The translation process was then implemented according to the guideline [40]. First, SASS was translated from English to Farsi by 2 experts in health education and health promotion and psychology. Then, the 2 Farsi versions obtained were compared, and after combining, a single Farsi version of SASS was obtained. In the next step, 2 bilingual experts performed backward translation from Farsi to English translated the Farsi version of the SASS questionnaire into English and compared it with the original version. No specific discrepancies were identified during these steps. Finally, the final English version was retranslated into Farsi by experts in education and health promotion and psychology, and the final Farsi version was created. After the initial forward and backward translation, the pre‐final version was reviewed by a panel of bilingual experts in psychology and public health, who assessed the conceptual equivalence and cultural relevance of each item. After the initial forward and backward translation, the pre‐final version was reviewed by a panel of bilingual experts in psychology and public health, who assessed the conceptual equivalence and cultural relevance of each item.

##### 2.2.1.4 Validation Process

In this phase, the face validity, content validity, and construct validity of the Persian version of the SASS questionnaire were evaluated.

##### 2.2.1.5 Face Validity

Due to the fact that the SASS questionnaire is a standard questionnaire, the face validity was evaluated only qualitatively. In this way, 9 people of participants (Education level above bachelor's degree ‐ familiar with psychological issues) were asked to review the questionnaire and comment on the meaning and purpose of the items, the difficulty of the items, ambiguity in words or phrases, appropriateness of the items and misconceptions. In case of any of the above problems, the questionnaire was reviewed and the items were modified if necessary.

##### 2.2.1.6 Content Validity

The content validity of the SASS was evaluated employing quantitative and qualitative methods. In this way, 9 numbers of specialists in various fields of psychology and health education and health promotion were asked to review the questionnaire and comment on grammatical points, word selection and use of words, simplicity of items, and clarity of items, appropriateness of the Likert scale with the items, and allocation and placement of items. If there was a problem in any of the above, the questionnaire was revised and if necessary, the items were modified or removed.

Also, in order to evaluate the content validity quantitatively, Lawshe technique was applied. CVR (Content Validity Ratio) and CVI (Content Validity Index) were calculated [41]. CVR evaluates the necessity of each of the items with a 3‐point Likert scale (not essential = 1, useful but not essential = 2, and essential = 3), and CVI evaluates the relevancy of each of the items with a 4‐point Likert scale (totally relevant = 4, relevant = 3, somewhat relevant = 2, not relevant = 1). The acceptable rate for CVR is depends on the number of evaluators. Since the number of evaluators was 9 in this study, the rate more than 0.75 is acceptable for CVR [41]. Also, the rate of 0.78 and more is acceptable for CVI [42].

##### 2.2.1.7 Construct Validity

In this phase, construct validity was checked by CFA (Confirmatory factor analysis). AMOS software (version 24) was used to conduct the confirmatory factor analysis (CFA). Before running CFA, Mahalanobis statistical test was used to find outlier data. Then some data were removed. Then, kurtosis and skewness indices were used to check the normality of the data. Then, the factor loadings of the items were checked. According to Stevens, the factor loading should be greater than 0.4 [43]. Then, the goodness of fit indexes of RMSEA, IFI, GFI, χ2/df, CFI, AGFI, TLI, PNFI, and PGFI were used for evaluating the final model. The adequate rate of goodness of fit indexes are χ2/df less than 5, IFI more than 0.9, PNFI more than 0.5, CFI more than 0.9, GFI more than 0.9, PGFI more than 0.5, TLI more than 0.9, AGFI more than 0.9, and RMSEA less than 0.08 [44, 45, 46, 47].

##### 2.2.1.8 Reliability

In this study, the stability reliability (test‐retest) was evaluated by calculating Intraclass Correlation Coefficient (ICC) and the internal consistency reliability evaluated by calculating McDonald's omega coefficient and Cronbach's alpha coefficient. The acceptable range for Cronbach's alpha coefficient is more than 0.70 [48, 49]. In order to calculate the ICC, 24 of the participants completed the questionnaire and recompleted the questionnaire after 2 weeks. Then ICC was calculated and ICC > 0.80 is acceptable [50]. McDonald's omega coefficient was calculated using JASP Version 0.11.1 software and ICC indices and Cronbach's alpha were calculated using IBM SPSS V.24 software.

#### 2.2.2 Ethics Approval and Consent to Participate

This research **adhered to ethical guidelines** and was approved by the Ethics Committee of Mashhad University of Medical Sciences (Approval Code: IR. MUMS. REC.1402.190). Additionally, written informed consent was secured from every participant.

## 3 Results

### 3.1 Demographic Characteristics

In this study, 962 people finally completed the questionnaire, and 38 people's questionnaires were excluded due to incomplete completion or outlier data. In this research, the mean ( ± standard deviation) age of participants was 32.75 ( ± 12.91). Most of people were married (*n* = 552, 55.2%), female (*n* = 601, 60.1%), and had bachelor degree (*n* = 303, 30.3%) (Table 1).

**Table 1:** Frequency distribution of demographic characteristics (*n* = 962).

Variables		*n*	%
Sex	Male	353	35.3
	Female	601	60.1
	Missing data	46	4.6
Marital status	Married	552	55.2
	Single	363	36.3
	Divorced	28	2.8
	Missing data	57	5.7
Occupation	Student university	181	18.1
	Housewife	178	17.8
	Employed	199	19.9
	Retired	50	5.0
	Self‐employed	133	13.3
	laborer	57	5.7
	Unemployed	31	3.1
	School student	77	7.7
	Missing data	94	9.4
Education level	Illiterate	6	0.6
	Elementary school	22	2.2
	Middle school	37	3.7
	High school	125	12.5
	Diploma	263	26.3
	Associate degree	120	12.0
	Bachelor degree	303	30.3
	Master's degree or high degree	66	6.6
	Missing data	58	5.8
Received the information about mental health	Yes	733	73.3
	No	197	19.7
	Missing data	70	7.0

### 3.2 Face and Content Validity

In the stage of qualitative face and content validity, based on the recommendations of participants and experts only six items were modified and simple and synonyms equivalent words were used. S‐CVI/Ave was calculated for all items of SASS and was 0.963.

### 3.3 CFA

**Figure 1:**
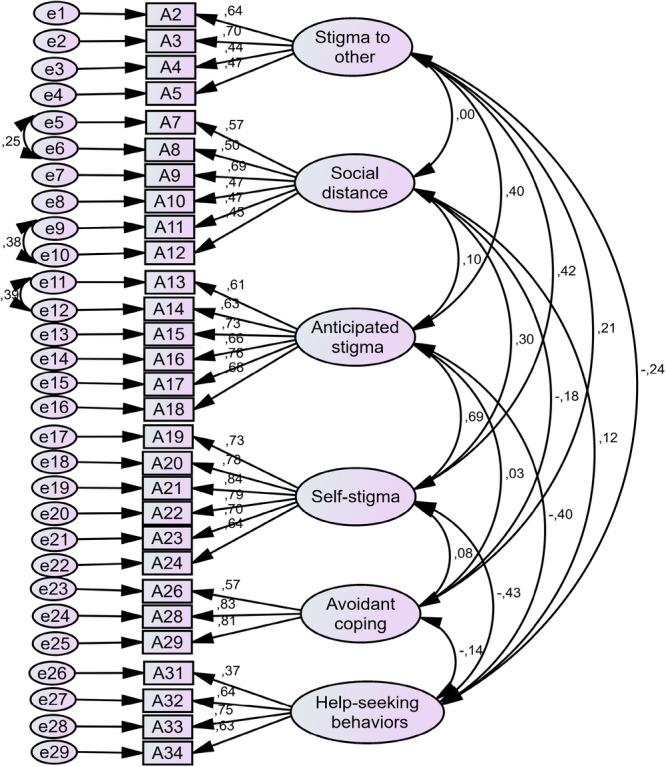
Standardized parameter estimates for the factor structure of the Persian version of SASS.

In this part, the scale with 6 factors and 36 items were surveyed. The factor loading of seven items of SASS were low than 0.3 and to improving goodness‐of‐fit indexes and confirmed the final model, seven items were deleted (*A1, A6, A25, A27, A30, A35 and A36*) (Table 2). Based on the results of goodness‐of‐fit indexes in Table 3 (e.g.,: X2/df =3.325, RMSEA = 0.049, CFI = 0.912, GFI = 0.920, IFI = 0.912, TLI = 0.901, PGFI = 0.759), SASS was approved with 29 items and 6 factor of Self‐stigma (6 items), Stigma to others (4 items), Anticipated stigma (6 items), Avoidant coping (3 items), Social distance (6 items) and Help‐seeking behaviors (4 items) (Table 2, Figure 1).

**Table 2:** Factor loadings of the Persian version of SASS.

Factor	Items	Factor loading
Stigma to others	1. People with mental disorders are NOT really ill and should just get on with things	Deleted
	2. Employees suffering from mental disorders are less reliable than other employees	0.644
	3. People with mental disorders are weak	0.695
	4. People with mental disorders should just “snap out of it”	0.442
	5. People with mental disorders cannot live good, rewarding lives	0.467
	6. People with mental disorders are NOT really ill	Deleted
Social distance	7. I'm good at talking to people with mental health problems	0.649
	8. I am comfortable when around people with a mental disorder	0.583
	9. I would feel comfortable discussing a colleague's mental health problem with them	0.670
	10. If I were an employer, I would feel comfortable employing someone with a mental disorder	0.446
	11. If I had a mental health disorder and needed help, I would feel comfortable going to a therapist	0.435
	12. Having a mental disorder is nothing to be ashamed of	0.421
Anticipated stigma	13. If I had a mental disorder, I would worry other people would think that I am weak	0.609
	14. If I had a mental disorder, I would worry other people would avoid talking to me	0.634
	15. If I had a mental disorder, I would worry other people would think I was exaggerating my difficulties	0.726
	16. If I had a mental disorder, I would worry that other people might think that I was “not really ill”	0.656
	17. If I had a mental disorder, I would worry other people would think of me as a failure.	0.764
	18. If I had a mental disorder, I would worry other people would feel sorry for me or patronise me	0.679
Self‐stigma	19. If I had a mental disorder, I would feel ashamed	0.732
	20. If I had a mental disorder and I could not solve my own problems, I would feel bad about myself	0.784
	21. If I had a mental disorder, I would feel weak.	0.836
	22. If I had a mental disorder, I would feel like no one would want to get close to me	0.786
	23. I would feel a burden to my colleagues if I had a mental disorder	0.701
	24. I would feel a failure if I became mentally unwell	0.638
Avoidant coping	25. Drinking alcohol never helps when you are stressed	Deleted
	26. It's often best to ignore problems and hope they go away	0.565
	27. Taking illegal drugs can never help when you are stressed by something	Deleted
	28. I do my best not to think about my problems	0.826
	29. The best way to cope with problems is not to think about them	0.807
	30. Mental health problems are best tackled head on	Deleted
Help‐seeking behaviors	31. If I had a mental disorder, I would be happy to seek help from a mental health professional	0.366
	32. If I had a mental disorder, I would NOT feel comfortable telling my manager	0.637
	33. I would NOT tell anyone if I had a mental disorder in case they judge me	0.749
	34. I would NOT feel comfortable discussing my mental health problems with a colleague	0.636
	35. I am confident that I could ask for help if I had a mental health problem	Deleted
	36. It's best not to tell anyone about your mental health problems	Deleted

**Table 3:** The model fit indicators of the Persian version of SASS.

Goodness of fit indices	Confirmatory factor analysis	Adequate value
χ2	1193.566	—
df	359	—
X^2^/df	3.325	< 5
RMSEA	0.049	< 0.08
GFI	0.920	> 0.9
CFI	0.912	> 0.9
IFI	0.912	> 0.9
AGFI	0.903	> 0.9
TLI	0.901	> 0.9
PNFI	0.777	> 0.5
PGFI	0.759	> 0.5

### 3.4 Reliability Assessment

The values of 0.911 and 0.925 were calculated for Cronbach's alpha coefficient and McDonald's omega coefficient all items of SASS. Also, the value of 0.677 was calculated for ICC of all items of SASS. Based on the results, only factor of Help‐seeking behaviors had week Cronbach's alpha coefficient but the McDonald's omega coefficient of this factor was more than 0.6 and had acceptable value. Cronbach's alpha coefficient and McDonald's omega coefficient of other factors were more than 0.6 and had acceptable value. Also, only the ICC of factor of Self‐stigma was weak and other factors had the acceptable value. Finally, the values of Cronbach's alpha coefficient, McDonald's omega coefficient, and ICC of SASS were acceptable, except one of the subscales (help‐seeking behaviours), which had inadequate reliability and is recommended not to be used (Table 4).

**Table 4:** The reliability of the Persian version of SASS.

Factors	Item	Range of score	Cronbach's alpha coefficient	McDonald's omega coefficient	Intraclass correlation coefficient (ICC)	95% Confidence Interval		*p*‐value
						Lower Bound	Upper Bound	
Stigma to others	4	0–16	0.778	0.743	0.838	0.629	0.930	< 0.001
Social distance	6	0–24	0.815	0.833	0.910	0.795	0.961	< 0.001
Anticipated stigma	6	0–24	0.904	0.907	0.725	0.367	0.881	< 0.001
Self‐stigma	6	0–24	0.964	0.966	0.484	‐0.088	0.767	0.038
Avoidant coping	3	0–12	0.714	0.770	0.810	0.413	0.889	< 0.001
Help‐seeking behaviors	4	0–12	0.558	0.650	0.744	0.463	0.901	< 0.001
Total SASS	29	0–116	0.911	0.925	0.677	0.270	0.859	< 0.001

## 4 Discussion

This study aimed to evaluate the SASS scale psychometrics in the general population. In general, this questionnaire with 29 items and 6 factors of Self‐stigma (6 items), Stigma to others (4 items), Anticipated stigma (6 items), Avoidant coping (3 items), Social distance (6 items), and Help‐seeking behavior (4 items) was approved. Results showed that the questionnaire had acceptable reliability except one of the subscales (help‐seeking behaviours), which had inadequate reliability and is recommended not to be used. Seven items compared to the original version after confirmation factor analysis were deleted. The exclusion of these items appeared consistent with Iran's prevailing cultural norms, where alcohol use is prohibited and public attitudes toward mental illness and its treatment remain largely pessimistic. Cronbach's alpha for all Persian SASS factors, except for the help seeking behavior factor was higher than the English version of SASS [21]. This discrepancy may be attributed to the higher homogeneity of our study sample compared to the original English version. The results showed that while the validity is good, the test‐retest reliability is moderate. This pattern may occur because questionnaires assessing psychological states (e.g., anxiety, stigma, mood) measure inherently labile constructs that naturally fluctuate over time. Thus, even when using a valid instrument, only moderate test‐retest reliability would be expected.

In this study, the first factor was “Stigma to others”, which was confirmed with 4 items. The Stigma to others refers to the negative attitudes and behaviors of individuals about those who have psychological problems [51]. People who have no psychological problems usually have a more humiliating attitude towards people with problems, and this makes people with mental problems less likely to seek help. In Eisenberg study, the high levels of Stigma to others was related to low levels of seeking help in college students [12]. In this factor, two items were removed due to low factor loadings. This could indicate that the concept of denying the legitimacy of mental illness (e.g., “People with mental disorders are NOT really ill”) is not a strong indicator of stigmatizing attitudes in this sample, potentially due to varying levels of mental health literacy or a cultural tendency to normalize rather than pathologize certain conditions. The meaning of both items is that people with mental illness are not really ill and that mental disorders are not considered diseases. This could have several possible reasons. First, undoubtedly, people's avoidance of considering, for example, depression as a real disease could stem from stigmatization. But second, some participants may not have a stigmatization attitude towards mental illness and have a kind of normalization attitude towards mental illness and therefore do not consider diseases such as depression, anxiety, etc. as diseases. And third, given that a significant percentage of our participants had less than a bachelor's degree, low mental health literacy may have caused this type of response, meaning that our participants may easily consider diabetes and hypertension as diseases, but due to low mental health literacy, they may not consider anxiety, depression, etc. as diseases because they are not very familiar with mental illnesses, their symptoms, their treatment, etc.

The second factor to be approved was the “Social distance” factor, which was confirmed with 6 items. Social distance refers to the desire for people to interact with people with mental illness and is influenced by factors such as stereotypes and fear [52]. Stigma can create a social and emotional distance between people who have stigma toward others and the people who receive the stigma [53]. Therefore, the measurement of social distance is very important to investigate the state of stigma as an effective factor. Schomerus et al., in a study aimed at investigating the relationship between psychiatric treatments and intention of people to seek help in the German public population, showed that increasing the level of personal desire to social distance is associated with a reduction in the desire to seek psychiatric assistance [54].

According to the results of this study, the third factor that was approved was the “Anticipated stigma” factor, which was confirmed with 6 items. The predicted or perceived public stigma refers to the belief that if others are aware of the future psychological problem of a person, the person will face prejudice, discrimination and devaluation from others [55]. Research has shown that the Anticipated stigma is a strong predictor of self‐stigma and can lead to the avoidance or inadequate use of psychological assistance needed [56]. Lannin et al., in a study showed that the anticipated stigma leads to self‐stigma and thereby predicts helpful behavior [57].

The fourth factor approved in this study was “Self‐stigma”, which was confirmed by 6 items. Self ‐ Stigma occurs when people internalize stereotypes and negative prejudices related to having mental health problems and label themselves inferior. Self‐ Stigma beliefs are accompanied by resigning from social support, rejecting aid, avoiding treatment, quitting treatment and limited perspective for recovery [58]. Findings of a study showed that self‐stigma can worsen the condition of mental illness and lead to weaker health and quality of life. Self ‐stigma can reduce the sense of people valuable and finally can reduce the hope for achieving therapeutic goals. Findings a study showed that increasing self‐stigma reducing the intention to seeking the psychological helps among people with mental problems [55].

The fifth factor to be approved was “Avoidant coping”, which was confirmed with 3 items. This concept indicates the deliberate ignorance of mental health problems, or the use of incompatible coping strategies such as alcohol consumption or other substances, to prevent negative thoughts or emotions [59]. In Holubova study, there was a relationship between self‐stigma, coping strategies and quality of life in outpatient with neurotic spectrum disorders [60]. Another study on patients with schizophrenia, also showed that the use of negative coping strategies such as avoidance and resignation increase stigma in patients with schizophrenic spectrum disorders, and reducing the amount of negative coping strategies helps reduce self‐stigma [61]. Therefore, to measure the dimensions of self‐stigma, it is important to measure the coping strategies as an important factor. In this factor, three items were removed due to low factor loading. The removed items pertained to alcohol and illicit drug use, which are culturally sensitive and prohibited in Iran, likely leading to response bias. A primary factor is likely social desirability bias, where participants may have been reluctant to endorse these items truthfully to present themselves in a culturally acceptable manner, even if the behaviors were applicable. Furthermore, the negative phrasing of these items may have increased their cognitive complexity for respondents. In the study by Docksey et al., who examined the validity and reliability of the SASS in a population of British employees, just like our study, items 25, 27, and 30 had very low factor loadings, but the authors did not remove these items and adequate fit indices were reported [21].

According to the results of this study, the sixth factor to be approved was the “Help‐seeking behaviors” factor, which was confirmed with 4 items. DeBate et al., in a study aimed at impact stigma on the determinants of help seeking behaviors related to mental health among students, showed that stigma predicted the mental health help seeking behaviors [62]. Also, the results of Özaslan study confirmed that the self‐stigma has potential negative effects on adolescent help seeking behaviors related to mental health [63]. Unfortunately, this subscale did not have appropriate reliability indicators and it is recommended that it not be used or that it be re‐evaluated in psychometric studies.

## 5 Strengths and Limitation

One of the strengths was high sample size, and high sample size can decrease measurement bias for variables. Validity and reliability of SASS was checked among public community and SASS can be used for different groups. A limitation of this study is the reliance on self‐reported data, which may be subject to reporting errors. This includes the potential for social desirability bias, as previously noted, as well as potential variability in comprehension and literacy levels among participants, which could have influenced response accuracy. Most notably, the internal consistency (Cronbach's alpha) was suboptimal for several subscales, particularly the Help‐Seeking Stigma subscale. Consequently, the findings related to these specific subscales should be interpreted with caution. The Help‐Seeking Stigma subscale, in its current form, may not be suitable for clinical or individual decision‐making purposes. Unfortunately, this subscale did not have appropriate reliability indicators and it is recommended that it not be used or that it be re‐evaluated in psychometric studies. Furthermore, the Stigma to Others and Avoidant Coping subscales should also be used with caution due to their borderline reliability. Future research should focus on refining the items of these subscales to improve their reliability. An additional limitation of this study is the absence of ancillary measures to comprehensively assess convergent and discriminant validity. Furthermore, the sample was drawn from a single, relatively traditional city in Iran, which may limit the generalizability of the findings to more diverse or urban Persian‐speaking populations.

## 6 Future Directions

Building directly upon the limitations identified in this study, several future research directions are recommended. First, the psychometric properties of the Persian SASS should be evaluated in more diverse Iranian subpopulations, including clinical samples, various ethnic groups, and urban centers, to establish its broader applicability. Second, future work should focus on refining the items of the less reliable subscales (Help‐Seeking, Stigma toward others, Avoidant Coping) to enhance their internal consistency. Third, longitudinal studies are needed to track the evolution of stigma and self‐stigma, particularly in vulnerable populations such as children of parents with mental illness or family caregivers. Such research can inform the temporal stability and sensitivity to change of these psychometric tools, as well as their predictive validity for future mental health outcomes. Finally, expanding the scope of stigma assessment to include structural and intersectional stigma can provide a more comprehensive understanding of stigma experiences in Iran.

## 7 Clinical Implication

The findings of this study can contribute to a more accurate assessment of stigma, enabling more targeted interventions. For instance, elevated scores on the ‘Self‐stigma’ and ‘Anticipated stigma’ factors can directly inform therapeutic approaches such as Cognitive Behavioral Therapy (CBT) or Acceptance and Commitment Therapy (ACT) to challenge internalized negative beliefs and reduce fear of societal judgment. High scores on ‘Stigma toward others’ and ‘Social distance’ highlight the need for public anti‐stigma campaigns and educational programs aimed at the general population to foster empathy and reduce discrimination. Furthermore, identifying ‘Avoidant coping’ and barriers in ‘Help‐seeking behaviors’ can guide clinicians in developing coping skills training and proactively addressing obstacles to care. On a policy level, these findings underscore the need for implementing mental health literacy curricula in schools and creating supportive workplace policies that reduce stigma and encourage help‐seeking.

## 8 Conclusion

In general, the results of this study showed that SASS has acceptable psychometric characteristics in the general population in Iran. This tool provides numerous aspects of stigma on mental health problems, including Self‐stigma, Anticipated stigma, Stigma to others, social distance, Avoidant coping and Help‐seeking behaviors. Therefore, this tool provides a foundation for Broader analysis of stigma and mental health attitudes and it can be used to formulate clinical guidelines to provide appropriate and purposeful information to consumers of mental health. However, the SASS scale requires further research to confirm its validity and reliability in different subgroups.

## Author Contributions

A.J., M.N., H.T., M.G., and F.N. designed the study. A.J., H.T., M.G., M.N., and F.N. participated in the conception of the study. F.N. and A.J. managed and conducted the statistical analysis and interpreted the data. A.J., H.T. and F.N. wrote the first draft and A.J., M.G.h., H.T., M.N., and F.N. revised it to make the final manuscript. All authors have approved the final manuscript. All authors have read and approved the final version of the manuscript [H.T. and A. J.] had full access to all of the data in this study and takes complete responsibility for the integrity of the data and the accuracy of the data analysis.

## Funding

The authors have nothing to report.

## Ethics Statement

This study is based on a research project approved by Ethics Committee of Mashhad University of Medical Sciences with the code of ethics IR. MUMS. REC.1402.190. All procedures performed in this study were in accordance with the ethical standards of the institutional and/or national research committee and with the 1964 Helsinki declaration and its later amendments or comparable. Written Informed Consent was obtained from all participant. Also, for individuals under 18 years old, informed consent to participate in the study was obtained from the appropriate parents/legal guardians.

## Conflicts of Interest

The authors declare no conflicts of interest.

## Transparency Statement

The lead author Fatemehzahra Naddafi affirms that this manuscript is an honest, accurate, and transparent account of the study being reported; that no important aspects of the study have been omitted; and that any discrepancies from the study as planned (and, if relevant, registered) have been explained.

## Data Availability

The data that support the findings of this study are available from the corresponding author upon reasonable request.
